# Decolonization of hospital patients may aid efforts to reduce transmission of carbapenem-resistant Enterobacterales

**DOI:** 10.1017/ash.2023.303

**Published:** 2023-09-29

**Authors:** Brajendra K. Singh, Prabasaj Paul, Camden D. Gowler, Sujan C. Reddy, Rachel B. Slayton

## Abstract

**Background:** Multimodal approaches are often used to prevent transmission of antimicrobial-resistant pathogens among patients in healthcare settings; understanding the effect of individual interventions is challenging. We designed a model to compare the effectiveness of hand hygiene (HH) with or without decolonization in reducing patient colonization with carbapenem-resistant Enterobacterales (CRE). **Methods:** We developed an agent-based model to represent transmission of CRE in an acute-care hospital comprising 3 general wards and 2 ICUs, each with 20 single-occupancy rooms, located in a community of 85,000 people. The model accounted for the movement of healthcare personnel (HCP), including their visits to patients. CRE dynamics were modeled using a susceptible–infectious–susceptible framework with transmission occurring via HCP–patient contacts. The mean time to clearance of CRE colonization without intervention was 387 days (Zimmerman et al, 2013). Our baseline included a facility-level HH compliance of 30%, with an assumed efficacy of 50%. Contact precautions were employed for patients with CRE-positive cultures with assumed adherence and efficacy of 80% and 50%, respectively. Intervention scenarios included decolonization of culture-positive CRE patients, with a mean time to decolonization of 3 days. We considered 2 hypothetical intervention scenarios: (A) decolonization of patients with the baseline HH compliance and (B) decolonization with a slightly improved HH compliance of 35%. The hospital-level CRE incidence rate was used to compare the results from these intervention scenarios. **Results:** CRE incidence rates were lower in intervention scenarios than the baseline scenario (Fig. 1). The baseline mean incidence rate was 29.1 per 10,000 patient days. For decolonization with the baseline HH, the mean incidence rate decreased to 14.5 per 10,000 patient days, which is a 50.2% decrease relative to the baseline incidence (Table 1). The decolonization scenario with a slightly improved HH compliance of 35% produced a relative reduction of 71.9% relative to the baseline incidence. **Conclusions:** Our analysis shows that decolonization, combined with modest improvement in HH compliance, could lead to large decreases in pathogen transmission. In turn, this model implies that efforts to identify and improve decolonization strategies for better patient safety in health care may be needed and are worth exploring.

**Figure:**
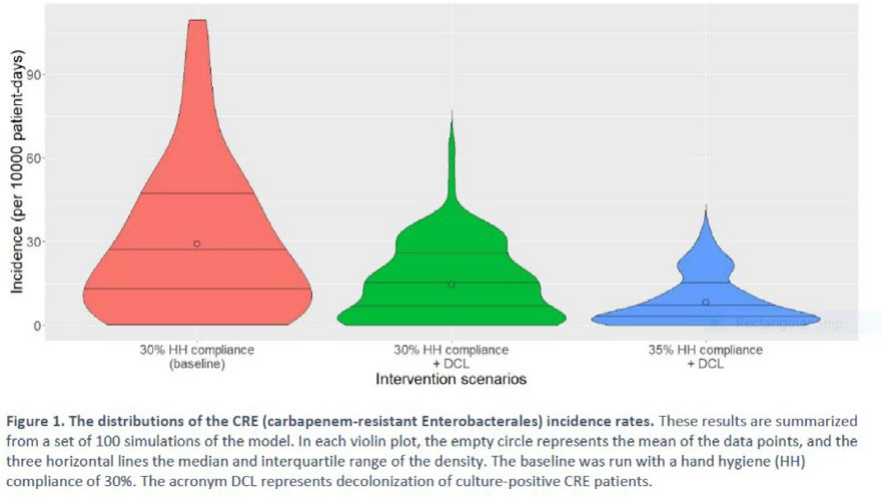

**Figure:**
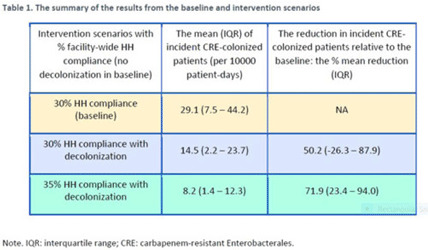

**Disclosures:** None

